# Activity-Based Proteomic Profiling of Deubiquitinating Enzymes in *Salmonella*-Infected Macrophages Leads to Identification of Putative Function of UCH-L5 in Inflammasome Regulation

**DOI:** 10.1371/journal.pone.0135531

**Published:** 2015-08-12

**Authors:** Evangel Kummari, Navatha Alugubelly, Chuan-Yu Hsu, Brittany Dong, Bindu Nanduri, Mariola J. Edelmann

**Affiliations:** 1 Department of Basic Sciences, College of Veterinary Medicine, Mississippi State University, Mississippi State, Mississippi, United States of America; 2 Institute of Genomics, Biocomputing and Biotechnology, Mississippi State University, Mississippi State, Mississippi, United States of America; Indian Institute of Science, INDIA

## Abstract

Although protein ubiquitination has been shown to regulate multiple processes during host response to *Salmonella enterica* serovar Typhimurium infection, specific functions of host deubiquitinating enzymes remain unknown in this bacterial infection. By using chemical proteomics approach, in which deubiquitinating enzymes were labeled by an active-site probe and analyzed by quantitative proteomics, we identified novel deubiquitinases in chicken macrophages based on their reactivity with the probe. Also, we detected down-regulation of UCH-L3, and USP4 as well as up-regulation of USP5 and UCH-L5 deubiquitinating enzymes in macrophages infected with *Salmonella* Typhimurium. We showed that decrease in either UCH-L5 activity, or in UCH-L5 protein amount in chicken and human macrophages infected or stimulated with LPS/nigericin, led to decreased IL-1β release. These data point towards a putative role of UCH-L5 in inflammasome regulation during *Salmonella* infection. Because inflammasome activation is important in innate resistance to these bacteria, one would expect that naturally occurring or therapeutically induced alteration in UCH-L5 activation would influence disease outcome and could represent a target for new therapeutic approaches.

## Introduction

*Salmonella enterica* serovar Typhimurium is a prevalent cause of zoonosis [1, 2] and it infects chickens, which is one of the most relevant vectors of this human foodborne disease. The processes related to bacterial entry, bacterial proliferation, or avoidance of bacterial killing by phagocytosis rely on host-pathogen interactions, in which protein post-translational modifications, such as ubiquitination play important roles [3]. *Salmonella* Typhimurium induces the formation of polyubiquitinated aggregates in epithelial cells and macrophages, and these polyubiquitinated aggregates increase autophagic flux, which enhances bacterial clearance [4]. The presence of these polyubiquitinated aggregates is regulated by a bacterial deubiquitinating enzyme (DUB), SseL, which reduces autophagic flux and this leads to increased bacterial replication [4]. Although there is clear modulation of polyubiquitination in *Salmonella*-infected cells, most DUBs that specifically regulate this modification in infection are unknown. Deubiquitinating enzymes are perturbed by other microbial pathogens, for instance to increase infection by blocking innate immunity [5]. Roles of some DUBs have been already described in other bacterial infections, such as OTUB1 [6, 7], UCH-L1 [8], USP7 [9], or CYLD [10–13]. The purpose of our study was to identify the host DUBs with altered activity and stability during *Salmonella* Typhimurium infection in chicken macrophages and describe their potential function in immune response to infection. By using activity-based ubiquitin probe followed by quantitative proteomics, we identified novel chicken DUBs and verified their activity on the basis of their reactivity with a ubiquitin probe. Moreover, we detected down-regulation of UCH-L3 and up-regulation of UCH-L5 (Ubiquitin C-terminal hydrolase 37; UCH37) activity in macrophages infected with *Salmonella* Typhimurium. We thereby demonstrated that chemical proteomics approach can be used to unambiguously quantify active enzymes in infected samples. We also showed that overexpression of UCH-L5 was associated with a significant increase in caspase-1 activity, while inhibition of UCH-L5 by selective inhibitor [14] or UCH-L5 knock-down led to decrease in inflammasome-dependent IL-1β release in chicken as well as in human macrophages during infection with *Salmonella* and during inflammasome activation by lipopolysaccharide (LPS) and nigericin. In summary, we demonstrated that UCH-L5 is an important enzyme whose activity is increased during infection with *Salmonella*, and that its increased catalytic activity leads to inflammasome activation and therefore more effective immune response.

## Materials and Methods

### Reagents

Chemicals were purchased from Sigma-Aldrich (St Louis, MO, USA), unless indicated otherwise. The antibodies used in this study were the following: goat anti-UCH-L5 antibody (Santa Cruz Biotechnology, USA), mouse anti-β-actin antibody (Sigma-Aldrich, USA), Mouse anti-HA antibody (Sigma- Aldrich, USA), mouse anti-GAPDH antibody (Thermo Scientific, USA), mouse anti-FLAG M2 antibody (Sigma Aldrich, USA), rabbit anti-UCH-L3 antibody (Cell Signaling, USA), rabbit anti-IL-1β (Abcam, USA), rabbit anti-IL-1β (Santa Cruz Biotechnology, USA).

### Cell and bacterial culture

HD11 cells (a gift from Dr. M. Parcells, [15]) were maintained in Advanced Dulbecco’s modified Eagle’s medium (DMEM) containing 2.5% fetal bovine serum, 1% Glutamax (Gibco) and 100 μg/mL streptomycin and penicillin in a humidified atmosphere of 5% CO2 at 37°C. The cells were maintained in culture by using Tryple Select (Gibco, USA).

THP-1 cells (ATCC, USA) were grown in RPMI 1640 medium (Gibco, USA) supplemented with 10% FBS and 100 μg/mL streptomycin and penicillin (Pen-Strep) in a humidified atmosphere of 5% CO2 at 37°C. For differentiation an activation of THP-1 cells, 100nM phorbol 12-myristate 13-acetate (PMA) was used and cells were differentiated for 24 hours before the experiments.

THP-1 control (null) and NLRP3-deficient cells were purchased from Invivogen Inc. and were cultured in RPMI 1640 medium (Gibco, USA), supplemented with 10 mM HEPES, 10% heat-inactivated fetal bovine serum, 100 μg/ml Normocin, Pen-Strep (50 U/ml-50 μg/ml). Selective antibiotic, Hygromycin B Gold (200 mg/ml), was added to cell culture medium following every other passage. These cells were differentiated with PMA as described above.

*Salmonella enterica* serovar Typhimurium wild-type ATCC 14028 [16] (a gift from David Holden, Imperial College London, UK) was cultured in Tryptic Soy broth (Fisher Scientific, USA) at 37°C at 200 rpm.

### Bacterial infections

Macrophage cells were incubated for 24 hours after plating, washed with phosphate buffered saline (PBS) and incubated in growth medium containing no antibiotics for 30 to 60 minutes before infection. *Salmonella enterica* serovar Typhimurium 12023 (ATCC 14028) overnight cultures cultivated at 37°C were diluted 1:20 and incubated further for at 37°C until OD_600_ was 0.5.

Bacteria were washed in PBS and used to infect cells (MOI of 50:1) for 1 h at 37°C. If longer time (infection for ~18 hours) was needed to access the protein level (or activity) of deubiquitinating enzymes and cell death, the cells were washed three times with PBS and incubated for 1 h at 37°C in growth medium supplemented with 10% fetal bovine serum and 100 μg/mL gentamicin. Medium was then changed to one containing 16 μg/mL gentamicin and incubated for 14 hours.

### Activity-based profiling of DUBs

HD11 macrophages were infected for 0 and 18 hours (or as indicated in figures) with *Salmonella* Typhimurium at multiplicity of infection (MOI) of 50:1. Cells were lysed in 0.1% NP-40, 150 mM NaCl, 20 mM CaCl_2_, 50 mM Tris pH 7.4 buffer containing 1 mM phenylmethanesulfonyl fluoride (PMSF). Samples containing equal amounts of protein were subjected to the enzymatic reaction with the ubiquitin vinyl sulfone HA-tagged probe (Ub-VS-HA; Boston Biochem, USA) at a ratio of ~1:200 for 45 minutes at 37°C. The proteins were separated by SDS-PAGE and subjected to anti-HA western blotting to visualize the active DUBs. For proteomics analysis, macrophages were infected for 0 and 18 hours with *Salmonella* Typhimurium (MOI of 50:1). After infection, the protein content was obtained and 8 mg protein per sample was subjected to the enzymatic reaction with the Ub-VS-HA probe as described above and immunoprecipitated as described in the section below.

### Immunoprecipitation, chloroform/methanol precipitation and tryptic digestion

For immunoprecipitation, 8 mg of protein per sample obtained as described in the section above was diluted in NET buffer (50 mM Tris, 5 mM EDTA, 150 mM NaCl, 0.5% NP-40, pH 7.4) to a protein concentration of 1 mg/ml, pre-cleared with agarose-coupled Protein A beads (Sigma-Aldrich) for 1 hour at 4°C, and immunoprecipitated with 60 μl of anti-HA Agarose Conjugate (Sigma-Aldrich) for 14 hours at 4°C. The resin was washed four times with NET buffer and the elution was done for 20 minutes by using 0.3 ml of 100 mM glycine pH 2.5. The eluted proteins were precipitated by using chloroform and methanol, in which 200 μl total volume of sample was used, to which 600 μl methanol and 150 μl chloroform was added and vortexed, followed by addition of 450 μl MilliQ-H_2_0 and centrifugation at room temperature for 1 minute at 14,000 rpm. The upper aqueous phase was discarded. 450 μl methanol was added to the lower phase and interphase, vortexed and centrifuged at room temperature for 1 minute at 14,000 rpm. The supernatants were removed and the pellets were resuspended in 0.1 M Tris pH 7.8 containing 6M Urea and then subjected to tryptic digestion, which was done exactly as we previously published [17]. Alternatively, the samples were resolved on SDS-PAGE and subjected to silver staining by using Pierce Silver Stain for Mass Spectrometry kit (Fisher Scientific) according to the manufacturer’s instructions, followed by an excision of gel bands and tryptic digestion, which was done exactly as we described previously [6]. The peptides were then analyzed by HPLC-MS/MS using an LTQ Velos instrument (Thermo Scientific, USA) as we described previously [18]. See S1 File for more information regarding the data analysis.

### DUB inhibitor studies

For inhibition of UCH-L5 activity, we used b-AP15 (#662140, Millipore), which was suspended in Dimethyl sulfoxide (DMSO). Appropriate dilutions (as described in figures) were prepared in growth medium directly before use and added to cells. Control cells were treated with DMSO only, and the concentration of DMSO was below 0.05% in all cases.

### Cell viability measurement

To measure cell viability, we used PrestoBlue Cell Viability Reagent (Life Technologies). HD11 cells were grown in 24-well plates, and after appropriate treatment (as described in figures), PrestoBlue Reagent was added directly to cells in culture medium according to the manufacturer’s instructions, and incubated for 1–2 hours at 37°C. To correct for background fluorescence, a sample containing only cell culture media was included. The fluorescence was then measured at 560 nm excitation/590 nm emission by using SPECTRAmax M5 (Molecular Devices).

### Transient intracellular expression of chicken UCH-L3 and UCH-L5 proteins

Please refer to the S1 File.

### Knock-down of UCH-L5 in THP-1 macrophages

THP-1 cells were differentiated into macrophages (in duplicate per sample type) onto 24-well plate, 24 hours prior to the transfection. The nucleofection was done using UCH-L5 siRNA or negative control siRNA (Qiagen, USA), which was diluted in AD2 solution (Lonza Inc.). The nucleofection solution containing siRNA was added onto the cells, the dipping electrode was immersed and the EH 100 program in the Nucleofector 4D instrument (Lonza) was applied. After the nucleofection was complete, growth medium containing 20% FBS was added and cells were incubated for 24 hours prior to infection with *Salmonella* Typhimurium, MOI 50:1 for 1 hour. The cells were lysed and obtained proteins were resolved on SDS-PAGE and subjected to western blotting (anti-UCH-L5, anti-GAPDH for loading control). The cell culture medium was spun down for 5 minutes at 500 x g, and the obtained supernatants were subjected to ELISA-based quantitation of IL-1β secretion as described below.

### Inflammasome activity studies

For the inflammasome activation studies, cells were infected (or not) with *Salmonella* Typhimurium wild-type at MOI 50:1 for 18 hours. The collected cells were resuspended in cell lysis buffer (25mM HEPES, 5mM EGTA, 5mM DTT, pH 7.5) and caspase-1 activity was measured by using Z-YVAD-AFC substrate (Enzo Life Sciences, USA, S1 File). For measurement of IL-1β release into cell medium, the cells were primed with LPS (1 μg/ml) for 4 hours, followed by treatment with 1 μM b-AP15 (or vehicle control, DMSO) for 15 minutes, and 10 μM nigericin for 1 hour. The cell pellets were collected for caspase-1 activity measurement, while cell culture media were spun down at 500 x g for 5 minutes and used for detection of chicken IL-1β. For measurement of IL-1β release into cell medium during infection with *Salmonella*, cells were treated with 1 μM b-AP15 or DMSO (vehicle control) for 60 minutes, infected with *Salmonella* Typhimurium (MOI 50:1) for times indicated in figures. Cell culture media were analyzed by western blotting using IL-1β antibody. For chicken IL-1β quantitation, we used ELISA assay (MyBioSource, Inc., USA) and western blot analysis. Supernatants from THP-1 cells treated or not treated with b-AP15 and infected or not infected with *Salmonella* were spun down for 5 minutes at 500 x g to remove any cells, and subjected to ELISA-based quantitation of human IL-1β (R&D systems, USA), which was performed according to the manufacturer’s instructions.

### Statistical analysis

The statistical analysis was done by using Student t-test in JMP Pro 11.0.0 (SAS Institute Inc., USA) or GraphPad Prism 6 (GraphPad Software, Inc., USA). For the proteomics data, the Fisher’s exact test was performed using the proteomics analysis software Scaffold Proteome, which has in-built appropriate statistical tests for large data sets (Scaffold, Proteome, USA). Graphs were generated in Excel (Microsoft Office, USA) and in GraphPad Prism 6 (GraphPad Software, Inc., USA).

## Results

### Activity profiling DUBs in *Salmonella* Typhimurium-infected cells by using a ubiquitin-specific active-site probe

**Fig 1:**
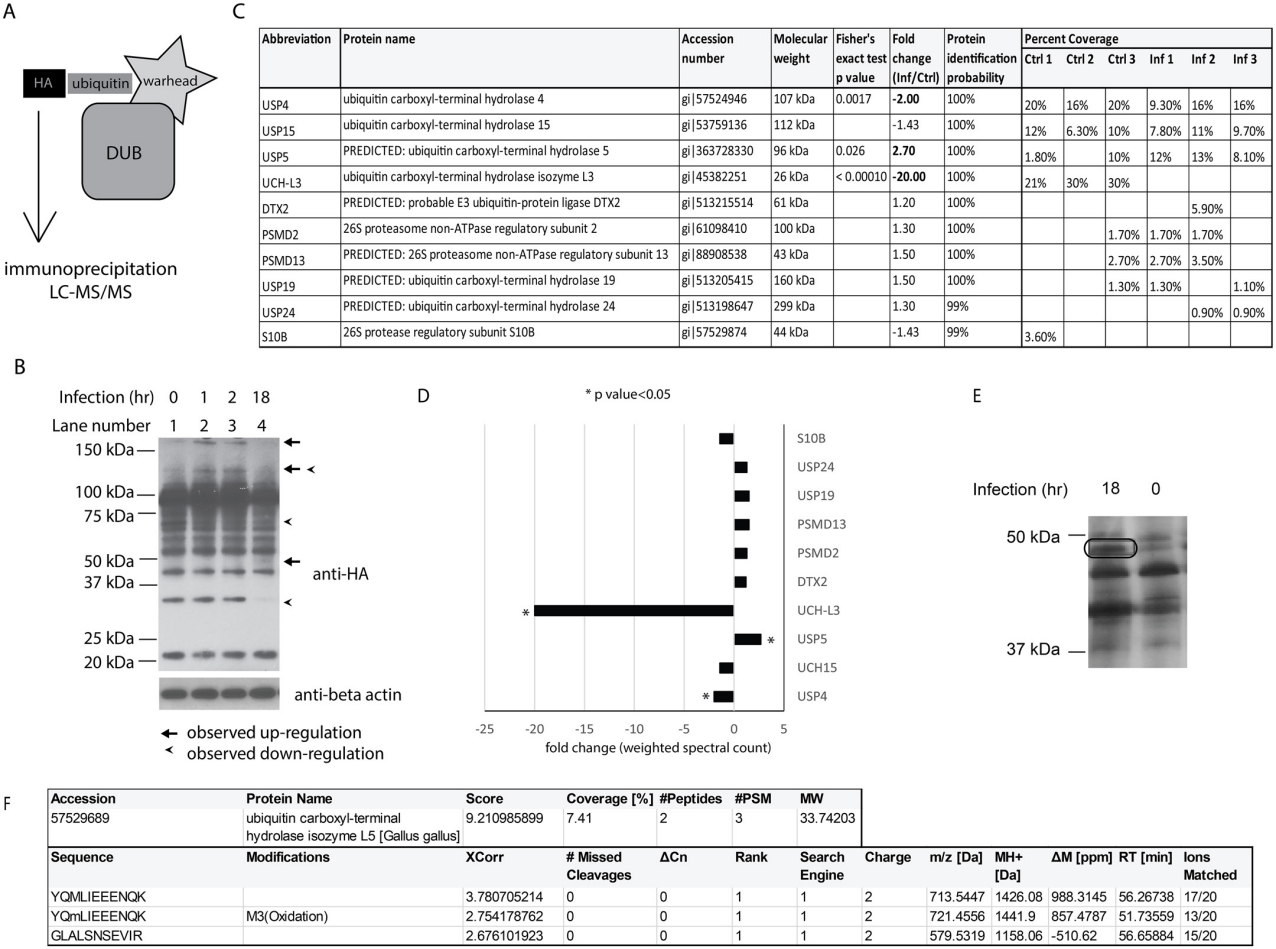
Activity profiling of DUBs in *Salmonella*-infected cells by using ubiquitin-specific active-site probe. **(A). DUB probe anatomy.** Ubiquitin-specific active-site probes consist of a retrieval element (tag), such as HA, which enables visualization and purification of a probe-bound DUB, as well as a ubiquitin molecule used for site recognition, and warhead (such as hereby used vinyl sulfone), which is a reactive group that interacts with DUB’s cysteine by formation of a thioester. **(B). Activity profiling by ubiquitin-specific active-site probe in *Salmonella*-infected macrophages.** HD11 macrophages were infected in duplicate for 0, 1, 2 and 18 hours with *Salmonella* Typhimurium at multiplicity of infection (MOI) of 50:1. After infection, the protein content was obtained and subjected to the enzymatic reaction with Ub-VS-HA probe. The proteins were separated by SDS-PAGE and subjected to anti-HA western blotting to visualize the active DUBs. One representative blot is shown. **(C, D)**. **Identification of DUBs regulated in HD11 macrophages during *Salmonella* infection by chemical proteomics.** HD11 macrophages were infected for 0 and 18 hours with *Salmonella* Typhimurium at MOI of 50:1. After infection, the protein content was obtained and subjected to the enzymatic reaction with the Ub-VS-HA probe. Probe-bound DUBs were immunoprecipitated by using anti-HA agarose and subjected to tryptic digestion. The peptide mixtures were then analyzed by quantitative proteomics (HPLC-MS/MS). The table shows names of the identified proteins, their accession numbers (NCBI), molecular weight, Fisher’s exact test p-values, fold change (calculated from the weighted spectral count in infected versus uninfected samples), protein identification probabilities as well as percent of protein sequence coverage [%] in individual replicas. Only the DUBs identified with high confidence are shown (C). A graph displays fold change (calculated using weighted spectral count) of DUBs and other relevant ubiquitin-binding proteins in infected versus uninfected samples (D). The abbreviations refer to the names of proteins from the table (C). **(E-F). Identification of UCH-L5 upregulated in infected HD11 macrophages upon infection.** Immunoprecipitated DUBs obtained from uninfected and infected HD11 macrophages as described in (C) were resolved on SDS-PAGE and subjected to silver staining prior to band excision and tryptic digestion of the indicated band. The identified DUB corresponded to chicken UCH-L5 (F). Accession, protein name, protein score, protein sequence coverage [%], number of identified peptides and Peptide spectrum matches (PSMs) as well as expected molecular weight are shown for the identified protein. For each one of three UCH-L5 peptides, sequence, modification, XCorr value, number of missed cleavages, delta Cn value, peptide rank, search engine rank, peptide charge, molecular weight of a precursor ion and molecular weight of the calculated singly charged peptide are shown, as well as delta mass [ppm], retention time [minute] and number of ions matched.

Since it is known that polyubiquitination of proteins is regulated during *Salmonella* infection [3] yet the enzymes regulating deubiquitination (DUBs) in this infection are not known, we set out to discover which host DUBs are modulated during *Salmonella* infection. In order to profile a subset of DUBs, we used quantitative chemical proteomics approach. In this workflow, we utilized ubiquitin vinyl sulfone HA-tagged (Ub-VS-HA) probe, which contains ubiquitin modified at its C terminus with an electrophilic trap (vinyl sulfone) that reacts with active-site cysteine of DUBs [19], and this property allows for activity profiling of this enzyme group [5, 20] (Fig 1A). We infected chicken HD11 macrophages in duplicates for 0, 1, 2 or 18 hours with *Salmonella* Typhimurium at multiplicity of infection (MOI) of 50:1. After infection, the protein content was obtained by cell lysis, and the enzymatic reaction with the Ub-VS-HA probe was carried out. The active enzymes (i.e. covalently modified by an HA-tagged probe, which adds approximately 10 kDa to their molecular weight) were then separated by sodium dodecyl sulfate polyacrylamide gel electrophoresis (SDS-PAGE) and anti-HA western blotting was carried out to visualize the active DUBs (Fig 1B). This experiment indicated that there are DUBs, which change their activity (or abundance) during *Salmonella* infection. To discover the identities of these DUBs and perform label-free quantification, we used proteomic analysis, since many labeled DUBs have similar molecular weight and therefore migrate similarly on the SDS-PAGE, which decreases the resolution of this approach (Fig 1B). We performed large-scale culture of HD11 macrophages in triplicates infected with *Salmonella* for 0 and 18 hours. Quantitative proteomics of purified active DUBs and other proteins reacting with the Ub-VS-HA probe was done by using high-performance liquid chromatography in combination with tandem mass spectrometry (HPLC-MS/MS, LTQ). The quantitative analysis indicated up-regulation of USP5 and down-regulation of UCH-L3 and USP4 in *Salmonella*-infected cells (Fig 1C and 1D). We also identified several proteasome subunits (Fig 1C). Some predicted DUBs were not identified by this approach, such as a protein between 37 and 50 kDa, which was up-regulated as an effect of infection (faint band indicated by an arrow, Fig 1B; Lane 4). This was most likely caused by a high abundance of few DUBs that strongly reacted with the Ub-VS-HA probe (Fig 1B; 75 kDa-120kDa molecular weight range). Therefore we used an alternative technique, in which we resolved the immunoprecipitated DUBs on SDS-PAGE, followed by silver staining of the proteins that reacted with the probe and excision of the bands of interest, tryptic digestion and LC-MS/MS analysis. Silver staining of this gel [Fig 1E, in which also contaminating proteins were present, as opposed to the western blot shown (Fig 1B), in which only proteins reacting with anti-HA antibody were detected) indicated presence of a DUB, which in size corresponded to the up-regulated protein detected by a western blot (Fig 1B) in infected samples. It was identified by LC-MS/MS as UCH-L5 (gi|57529689) on the basis of three peptides (Fig 1F), all of which were missing from the uninfected control sample.

**Fig 2:**
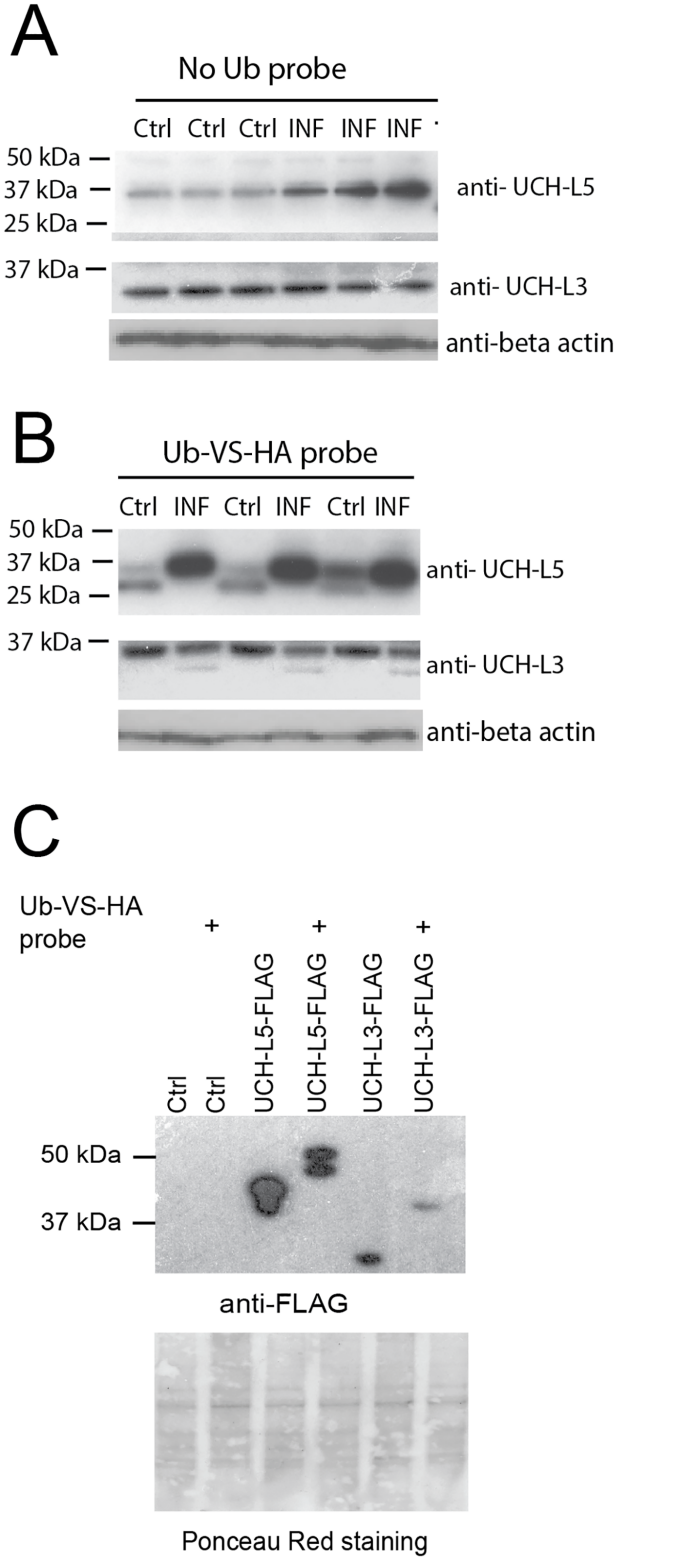
UCH-L5 is up-regulated while UCH-L3 is down-regulated in infected cells and both of these uncharacterized chicken DUBs are catalytically active. **(A-B). UCH-L5 and UCH-L5 DUBs are regulated in *Salmonella*-infected macrophages.** HD11 macrophages cells were infected as mentioned above in triplicates for 0 and 18 hours with *Salmonella* at MOI of 50:1, followed by cell lysis. The protein samples were separated by SDS-PAGE and anti-UCH-L3 and anti-UCH-L5 western blotting was done to demonstrate changes in protein level. The anti-β actin western blot was done as a loading control (A). Alternatively, these lysates were subjected to a reaction with the Ub-VS-HA probe (B) and processed as above. **(C)**. **Overexpressed chicken UCH-L3 and UCH-L5 are active DUBs.** FLAG-tagged UCH-L5 and UCH-L3 were overexpressed in HD11 macrophages and empty vector was used as a control. 24-hours past overexpression, the cells were lysed and equal amounts of proteins were incubated with Ub-VS-HA probe. The samples were analyzed by SDS-PAGE, followed by anti-FLAG western blotting to demonstrate expression and Ub-VS-HA probe binding to these DUBs. Ponceau Red staining of the membranes was used as a loading control.

To confirm these results by an alternative technique, we infected HD11 macrophages in triplicates for 0 and 18 hours with *Salmonella*, followed by cell lysis and reaction with the Ub-VS-HA probe (Fig 2A and 2B). Enzymes modified with the probe can be detected by western blotting by an upshift in molecular weight (Fig 2B) in comparison to the enzymes, which were not incubated with the probe (Fig 2A). This confirmed the up-regulation of UCH-L5 and down-regulation of UCH-L3 upon *Salmonella* infection. In case of UCH-L3 protein levels were comparable in infected and uninfected samples, but the level of active UCH-L3 was clearly down-regulated (Fig 2B). This indicates that UCH-L5 protein levels are modulated by either a pathogen or a host, and only the activity of UCH-L3 is changed during infection with *Salmonella*, which could be caused by changes in post-translational modifications of this enzyme [21].

### Confirmation of chicken UCH-L5 and UCH-L3 DUB activity

Chicken UCH-L3 and UCH-L5 are novel chicken DUBs (S1 Fig) and to confirm their activity, we synthesized these two genes and cloned them into an expression vector followed by overexpression in HD11 macrophages. We confirmed that the overexpressed chicken UCH-L3 and UCH-L5 are indeed DUBs and possess catalytic activity by using Ub-VS-HA probe (Fig 2C). Human UCH-L5 antagonizes substrate degradation in proteasomes [22], it interacts with proteasomes [23], and also has the capacity to support the substrate-induced activation of the proteasomal ATPases [24]. However, there is nothing known about chicken UCH-L5 function in proteasome regulation.

### UCH-L5 expression leads to an increase in caspase-1 activity

**Fig 3:**
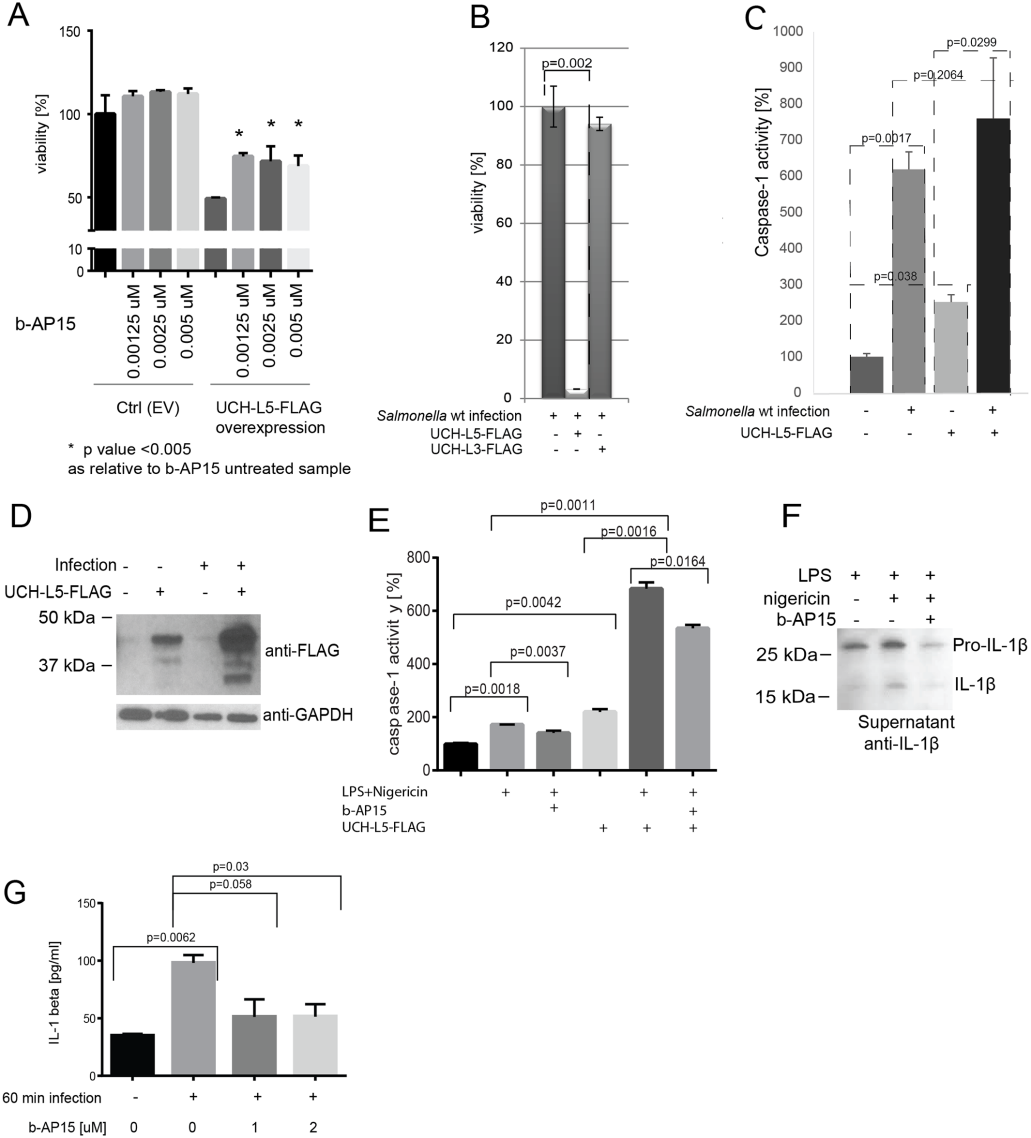
UCH-L5 regulates inflammasome activity in chicken macrophages. **(A-B). UCH-L5 overexpression leads to reduced cell viability in infected as well as uninfected HD11 macrophages, which depends on its catalytic activity.** UCH-L5 was overexpressed in HD11 macrophages and empty vector was used as a control (Ctrl). 24-hours past overexpression, the cells were treated with b-AP15 (UCH-L5 inhibitor) used at indicated concentrations for 18 hours. The cell viability was measured by Presto Blue assay (A). Alternatively, UCH-L5 was overexpressed in HD11 macrophages for 2 days prior to infection with *Salmonella* Typhimurium for 1 hour. The cell viability was measured by Presto Blue assay. The p-values were calculated by using Student T test. **(C) UCH-L5 overexpression leads to an increase in caspase-1 activity in HD11 macrophages.** UCH-L5 was overexpressed in HD11 macrophages (UCH-L5) and empty vector was used as a control (ctrl). 24-hours past overexpression, the cells were subjected to *Salmonella* infection at MOI of 50:1 for 18-hours (inf) or left uninfected. The caspase-1 activity was measured by using specific inhibitor. The p-values were calculated by using Student T test. **(D). Overexpressed UCH-L5 is increased in infected cells.** The protein cell lysates from (C) were analyzed by SDS-PAGE, followed by anti-FLAG western blotting to demonstrate UCH-L5 expression; anti-GAPDH western blotting was used as a loading control. **(E). Caspase-1 activity in HD11 macrophages is dampened upon b-AP15 inhibitor treatment.** UCH-L5 was overexpressed in HD11 macrophages (UCH-L5-FLAG) and empty vector was used as a control. 24-hours past overexpression, cells were primed with 1μg/ml LPS for 4 hrs followed by treatment with 1uM b-AP15 or vehicle control (DMSO) for 15 minutes to inhibit the activity of UCH-L5. Cells were then treated with 10μM nigericin for 1 hour to induce inflammasome. The cell pellets were collected and caspase-1 activity was measured by using Z-YVAD-AFC substrate. The p-values were calculated by using Student T test. **(F). Exposure of cells to b-AP15 inhibitor leads to decrease in IL-1β secretion in HD11 macrophages upon inflammasome activation.** The HD11 cells were seeded on 6-well plates (1x10^6^ cells, 3 replicates each) and primed with LPS (1μg/ml) for 4 hours followed by treatment with 1uM b-AP15 (or vehicle control, DMSO) for 15 minutes. Cells were then treated with 10uM nigericin (or not) for 1 hour to induce inflammasome. Media were collected and used for detection of chicken IL-1β by western blot. **(G). Exposure of chicken HD11 macrophages to b-AP15 inhibitor leads to decrease in IL-1β secretion during *Salmonella* infection.** HD11 cells were treated with 1μM b-AP15 or DMSO (vehicle control) for 60 min. They were then infected (or not) with *Salmonella* Typhimurium wild-type at MOI 50:1 for 60 minutes. Media were collected for ELISA-based quantitation of chicken IL-1β (MyBioSource, Inc., USA).

By using overexpression model of chicken UCH-L5 and b-AP15 inhibitor of UCH-L5/USP14 [14], which inhibited UCH-L5 at low molar concentration, but it did not significantly inhibit other DUBs in HD11 cells (S2 Fig), we observed the overexpression of UCH-L5 had a negative effect on cell viability, which depended on its catalytic activity as a DUB, since if UCH-L5 overexpressing cells were treated with b-AP15 inhibitor, the cell viability was partially restored (Fig 3A). This suggests that the DUB activity of chicken UCH-L5 is important in its function that leads to the cell death. Overexpression of UCH-L5 (but not overexpression of UCH-L3) for two days prior to the infection with *Salmonella* for one hour also lead to significant cell death (Fig 3B), while cell treatment with b-AP15 inhibitor prior to *Salmonella* infection led to slight increase in cell viability in comparison to vehicle-treated cells (S2 Fig).

Pyroptosis is an important cell death type that occurs during *Salmonella* infection, and since DUBs such as UCH-L5 might be relevant in regulation of inflammasomes [25], we tested caspase-1 activity in cells overexpressing UCH-L5. We transfected HD11 macrophages with UCH-L5 or negative control plasmid, and infected these cells with *Salmonella* Typhimurium prior to the measurement of the caspase-1 activity. The data indicate that overexpression of UCH-L5 leads to significantly increased caspase-1 activity. After 18 hours of infection the difference in caspase-1 activity in control and UCH-L5 overexpressing cells was not as significant, most likely because many cells were already not viable and only the end-point of the process was measured. Moreover, caspase-1 activity was significantly increased (over 6-fold) in infected cells as compared to uninfected cells (Fig 3C). The analysis of these cell pellets (Fig 3D) indicates that the overexpressed UCH-L5 was highly up-regulated post-infection in comparison to uninfected cells, similarly as endogenous UCH-L5 (Figs 1 and 2), which further validated our proteomic results.

Furthermore, to analyze effect of UCH-L5 expression and activity on caspase-1 activity upon inflammasome activation, we treated cells, which overexpressed UCH-L5-FLAG (or empty vector control cells) with LPS, followed by pre-treatment of cells by b-AP15 inhibitor. Cells were then treated with nigericin to up-regulate inflammasome activity [25]. Control cells treated with b-AP15 inhibitor of UCH-L5 had lower caspase-1 activity than control cells treated with vehicle control (DMSO; Fig 3E). Moreover, UCH-L5 overexpression led to significantly higher caspase-1 activity, even in cells not treated with LPS and nigericin. Finally, pre-treatment with b-AP15 inhibitor led to decrease in caspase-1 activity in UCH-L5 overexpressing cells.

### UCH-L5 inhibition down-regulates IL-1β release during inflammasome activation in macrophages

To test whether an inhibition of UCH-L5 in chicken HD11 macrophages leads to an increase in release of IL-1β cytokine due to increased inflammasome activity [25], we induced inflammasome activity by using LPS and nigericin, in cells pre-treated with b-AP15 inhibitor and quantified IL-1β by western blotting in cell culture medium. We noticed a significant decrease in pro-IL-1β and IL-1β released to cell culture medium as measured by western blotting (Fig 3F). Next to the processed IL-1β, pro-IL-1β has also been detected in the cell-free cell culture supernatant, and this has been previously documented for human pro-IL-1β, for example in cells exposed to ATP or anti-microbial peptides, which could be due to the loss of membrane integrity (reviewed in [26]). Also, *Salmonella* infection of chicken macrophages led to significant increase in secretion of IL-1β to cell culture supernatant, while b-AP15 compound inhibited secretion of pro-IL-1β in *Salmonella*-infected cells, as quantified by chicken IL-1β ELISA assay (Fig 3G).

**Fig 4:**
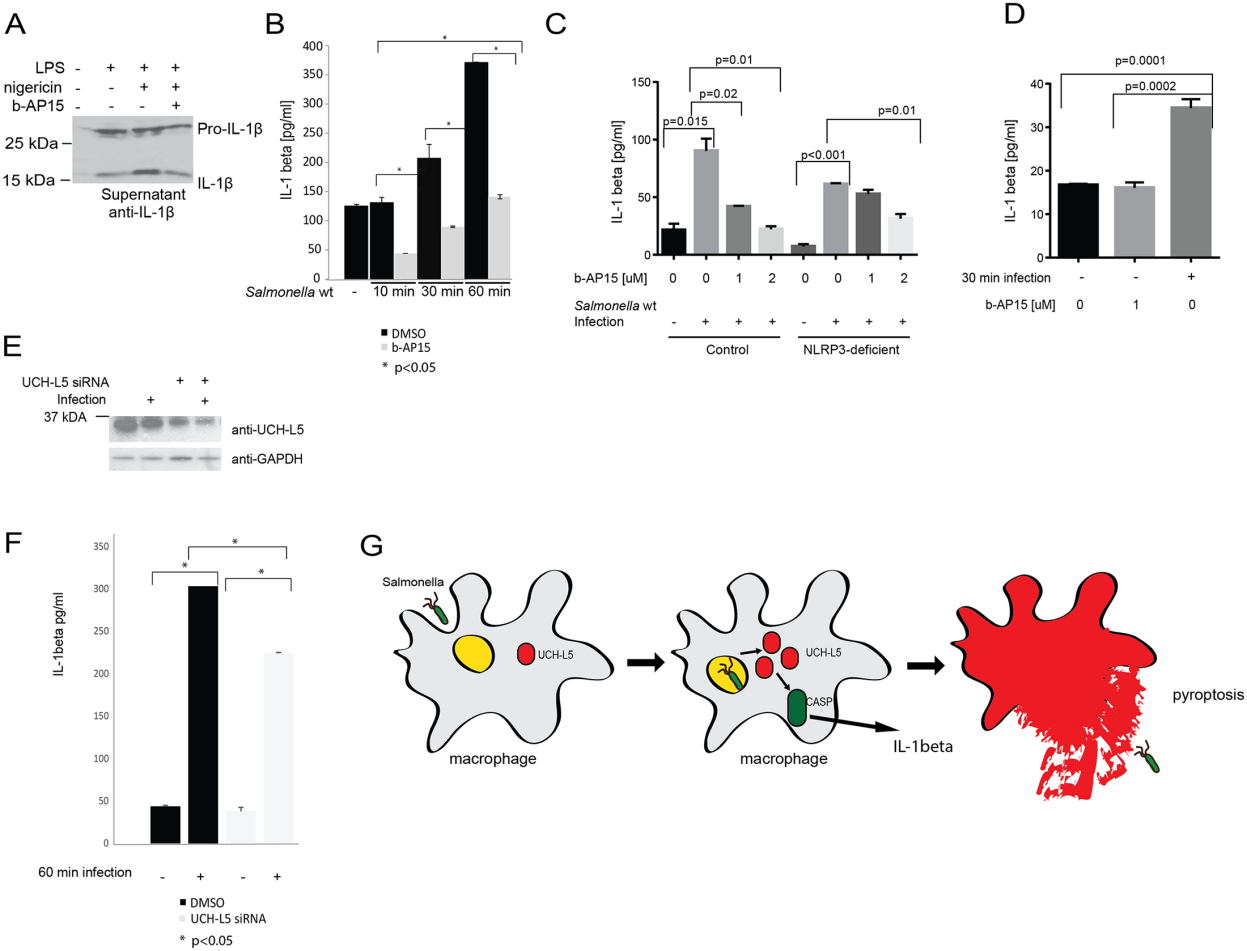
UCH-L5 regulates inflammasome activity in human macrophages. **(A). Exposure of THP-1 macrophages to b-AP15 inhibitor leads to decrease in IL-1β upon inflammasome activation.** The THP-1 cells were seeded on 6-well plates, treated with 100nM PMA for 24 hours and exposed to LPS treatment for hours, followed by treatment with 1μM b-AP15 or DMSO (vehicle control) for 60 min and treatment with nigericin for 60 minutes. Media were collected for western blotting of human IL-1β. **(B). Exposure of THP-1 macrophages to b-AP15 leads to decrease in IL-1β secretion upon *Salmonella* infection at different time points of infection.** The THP-1 cells were seeded on 24-well plates, treated with 100nM PMA for 24 hours and exposed to 1μM b-AP15 or DMSO (vehicle control) for 60 min. They were then infected with *Salmonella* Typhimurium wild-type at MOI 50:1 for indicated time points. Human IL-1β ELISA was used to quantify the amount of IL-1β I into the cell culture medium, which is shown in pg/ml. **(C). IL-1β secretion from *Salmonella*-infected control and NRLP3-deficient cells exposed and not exposed to b-AP15 inhibitor.** The THP-1 cells control cells as well as NLRP3-deficient cells (Invivogen Inc, USA) were seeded on 24-well plates, treated with 100nM PMA for 24 hours and exposed to b-AP15 or DMSO (vehicle control) for 60 min. They were then infected with *Salmonella* Typhimurium wild-type at MOI 50:1 for 1 hour. Human IL-1β ELISA was used to quantify the amount of IL-1β I into the cell culture medium, which is shown in pg/ml. **(D). The b-AP15 inhibitor does not affect IL-1β secretion from uninfected cells.** The THP-1 were treated with 100nM PMA for 24 hours and exposed to b-AP15 or DMSO (vehicle control) for 60 min. They were then infected or not with *Salmonella* Typhimurium wild-type for 30 minutes. Human IL-1β ELISA was used to quantify the amount of IL-1β I into the cell culture medium, which is shown in pg/ml. **(E-F). Partial knock-down of UCH-L5 in THP-1 macrophages leads to attenuation in IL-1β secretion in *Salmonella*-infected cells.** UCH-L5 was knocked-down in THP-1 macrophages by UCH-L5 siRNA (negative control siRNA was used as a control). After nucleofection was complete, new medium was added onto cells and cells were incubated for 24 hours prior to infection with *Salmonella* Typhimurium, MOI 50:1 for 1 hour. The cells were lysed and obtained proteins were resolved on SDS-PAGE and subjected to western blotting (Fig 4E; anti-UCH-L5, anti-GAPDH for loading control). IL-1β secretion to medium was quantified by ELISA (F). **(E). Model of UCH-L5’s effect on inflammasome activation in macrophages infected with *Salmonella* Typhimurium.**

UCH-L5 had similar effects in human macrophages. Human THP-1-derived macrophages were primed with LPS and pre-treated with b-AP15 inhibitor of UCH-L5 or DMSO vehicle control, before the induction of inflammasome by nigericin. In cells treated with b-AP15 inhibitor, there was a significant decrease in IL-1β release to medium in comparison to cells treated with vehicle control as shown by western blotting (Fig 4A). To quantify IL-1β release in response to *Salmonella* infection in THP-1-derived macrophages in a time-course experiment, human THP-1 macrophages were infected with *Salmonella* Typhimurium for 0, 10, 30 and 60 minutes and IL-1β release to cell culture medium was measured by ELISA-based quantitation. After 30 minutes of infection, there was a significant increase in IL-1β release to medium, which was even greater at 60 minutes of infection. However, in b-AP15-treated cells, these IL-1β levels were significantly attenuated (Fig 4B). To show that b-AP15 inhibitor led to attenuation of NLRP3 inflammasome activation during *Salmonella* infection, we used control THP-1 cells as well as NLRP3-deficient cells (Invivogen Inc, USA), which were activated with 100 nM PMA for 24 hours and exposed to b-AP15 or DMSO (vehicle control) for 60 min. These macrophages were then infected with *Salmonella* Typhimurium wild-type at MOI 50:1 for 1 hour, followed by IL-1β quantitation by ELISA assay (Fig 4C). The effect of b-AP15 on IL-1β release from NLRP3-deficient THP-1 macrophages infected with *Salmonella* was less significant in comparison to infected control THP-1 macrophages and only 2μM concentration of b-AP15 had a statistically significant negative effect on IL-1β release from these cells. Finally, we also showed that b-AP15 compound did not have any effect on IL-1β release from uninfected THP-1 macrophages (Fig 4D).

Moreover, we used Nucleofector 4D system (Lonza Inc.) to knock-down UCH-L5 from THP-1 macrophages by using siRNA (Qiagen Inc.), which was partially successful. We then infected these cells with *Salmonella* Typhimurium at MOI 50:1 for one hour (Fig 4E) and collected media, which were subjected to IL-1β level measurement by ELISA-based quantitation. Although the knock-down of UCH-L5 was only partial, we noted also partial attenuation in IL-1β release to medium from these cells in comparison to control cells (Fig 4F).

## Discussion

The role of ubiquitin modification in host responses to *Salmonella* Typhimurium infection has been widely recognized [3], yet little is known about the specific functions of enzymes regulating this post-translational modification in infection. Ubiquitination is used by the host but also by bacteria to influence the fate of the infection. Autophagic capture of *Salmonella* Typhimurium occurs via generation of a poly-ubiquitin signal around cytosolic bacteria, which leads to selective degradation by the autophagic machinery [27]. Formation of these *Salmonella*-dependent polyubiquitinated aggregates in host cells is negatively regulated by *Salmonella*’s own DUB, SseL. These polyubiquitinated structures were shown to be degraded by autophagy, which process is also down-regulated in the presence of SseL [4]. Another important function that the ubiquitin modification might have in *Salmonella* infection of host cells is pro-inflammatory cell death, or pyroptosis, which bypasses macrophage apoptosis during *Salmonella* infection and is dependent on caspase-1 activity [28]. Treatment with general DUB inhibitors (such as PR-619 and WP1130) has a negative effect on LPS/ATP-induced deubiquitination of NLRP3 and it also inhibits caspase-1 activation during *Salmonella* infection [29], which suggests that DUBs are involved in this process.

Activity-based chemical proteomics allows for monitoring of activity of multiple DUBs in infected cells, and we chose this technique to not only discover novel chicken DUBs that were previously uncharacterized, but also to unravel DUBs that are differentially regulated in *Salmonella* infection, which included USP4, USP5, UCH-L3 and UCH-L5. We validated quantification of only two of these proteins, UCH-L3 and UCH-L5 (Fig 2), mainly because of the limited availability of chicken-specific antibodies for the other DUBs. The function of UCH-L3 in infection remains to be uncovered, but in this study we concentrated on a putative effect of UCH-L5 expression on the host response to infection.

Since some DUBs might be involved in inflammasome assembly, although no direct effect of DUBs on caspase-1 activity was identified in *in vitro* studies [25], we tested whether chicken and human UCH-L5 can regulate pyroptosis during *Salmonella* infection as well as upon LPS/nigericin-triggered inflammasome activation. We indeed observed that overexpression of UCH-L5 leads to a significant increase in cell death, which is dependent on its catalytic activity (Fig 3A and 3B), and that its overexpression can lead to an increase in caspase-1 activity in macrophages (Fig 3C and 3E), while chemical inhibition of UCH-L5 leads to a decrease in caspase-1 activity (Fig 3E). This effect might be caused by direct deubiquitination of important components of inflammasome, which remain unknown. Although the chemical inhibitor used in this study, b-AP15, is capable of inhibition of another DUB, USP14, and the effect of USP14 on inflammasome activity cannot be completely ruled-out, we showed that both overexpression of UCH-L5 followed by chemical inhibition, as well as knock-down of UCH-L5 by specific siRNA had consistent effects on inflammasome activation (Figs 3A–3E, 4E and 4F). This UCH-L5-mediated modulation of caspase-1 activation limits IL-1β secretion into the culture medium upon *Salmonella* Typhimurium infection or inflammasome activation in chicken macrophages (Fig 3) as well as in human macrophages (Fig 4), which possibly is caused by UCH-L5 deubiquitinating some of the components of inflammasomes, such as caspase-1 or NLRP3, which needs to be tested further.

Interestingly, *Salmonella* Typhimurium inhibits its flagellin expression during infection of macrophages to replicate and not allow for the inflammasome to detect its presence. However, increased expression of UCH-L5, which we observed during infection, could lead to an increased caspase-1 activity in macrophages, which would then promote cell death (Fig 3A and 3B) and release of the bacteria from macrophages. This is consistent with previous published studies, which suggest that *Salmonellae* expressing flagellin are killed via a mechanism, that involves at least two steps: 1) pyroptotic death of macrophages, which results in externalization of the bacteria that were inside the macrophages, 2) uptake of bacteria by neutrophils and subsequent neutrophil-mediated killing (Fig 4E, [30]).

Caspase-1 activity is induced by *Salmonella* with the help of NOD-like receptors, NLRP3 and NLRC4, which recruit ASC and caspase-1, leading to pro–IL-1β processing [31]. Components of the inflammasome, such as ASC, have been shown to be ubiquitinated, leading to a recruitment of the autophagic adaptor p62, which assists in their delivery to autophagosomes [32]. NLRP3 is also polyubiquitinated and it has been shown that it can be deubiquitinated by BRCC3 [33]. Since the catalytic activity of UCH-L5 is necessary for its function in inflammasome activation (Figs 3 and 4; [25]), it is possible that it regulates NLRP3 or caspase-1 poly-ubiquitination, thus leading to an increase in caspase-1 activity, which will be further investigated.

To conclude, in this study we demonstrated that chemical proteomics can be applied to infectious diseases in order to discover novel enzymes regulated in bacterial infection. We identified new chicken DUBs on the basis of the reaction with the Ub-VS probe and characterized USP4, USP5, UCH-L3 and UCH-L5 as DUBs regulated in infection. Furthermore, by functional studies we demonstrated that UCH-L5, which activity is up-regulated in the host during *Salmonella* infection, might constitute an important pro-inflammatory deubiquitinase, which leads to an increased inflammasome activation and regulates secretion of IL-1β. Pro-inflammatory cytokines are required for *Salmonella* clearance and regulation of their production or processing constitutes an important component of the host response.

## Supporting Information

S1 File Supplementary methods. (DOCX)

S1 Fig Sequence alignment analysis of UCH-L3 and UCH-L5. (TIF)

S2 Fig Inhibition of UCH-L5 activity in HD11 cells. (TIF)

S1 Table List of primers used in PCR amplification. The restriction enzyme digestion sites are represented as the bolded letters, including HindIII (AAGCTT) and EcoRI (GAATTC).
(DOCX)
