# Correction: Activity-Based Proteomic Profiling of Deubiquitinating Enzymes in *Salmonella*-Infected Macrophages Leads to Identification of Putative Function of UCH-L5 in Inflammasome Regulation

**DOI:** 10.1371/journal.pone.0138635

**Published:** 2015-09-15

**Authors:** Evangel Kummari, Navatha Alugubelly, Chuan-Yu Hsu, Brittany Dong, Bindu Nanduri, Mariola J. Edelmann

The following information is missing from the Funding section: This study was supported by a Biotechnology Risk Assessment Grant Program competitive grant No. 2015-67016-22939 from the National Institute of Food and Agriculture and Agricultural Research Service.
