# Supplementary material for: Activity-Based Proteomic Profiling of Deubiquitinating Enzymes in Salmonella-Infected Macrophages Leads to Identification of Putative Function of UCH-L5 in Inflammasome Regulation
Source: PLoS One. 2015 Aug 12;10(8):e0135531. doi: 10.1371/journal.pone.0135531 (PMC4534353; doi:10.1371/journal.pone.0135531)
Supplement: S1 File — (DOCX) [file pone.0135531.s001.docx]

**Supporting Information (SI)**

Supplementary figure legends

**S****1 Fig. Sequence alignment analysis of UCH-L3 and UCH-L5.** The deduced amino acid sequences of UCH-L3 and UCH-L5 proteins cloned from HD11 cells were compared with sequences of UCH-L3 and UCH-L5 proteins from human, cow, mouse, rat, and chicken obtained from the GenBank database (http://www.ncbi.nlm.nih.gov/). The ClustalX was used to generate multiple sequence alignments. Cysteine peptidase C12 domain and catalytic site residues are indicated.

**S2 Fig. Inhibition of UCH-L5 activity in HD11 cells. (A).** HD11 macrophage cells were treated with two different amounts of b-AP15 inhibitor (as indicated in the figure) for 18 hours, followed by cell lysis, Ub-VS-HA probe reaction, SDS-PAGE and anti-HA western blotting to show general profile of DUBs affected by this DUB inhibitor. Anti-UCH-L5 western blotting was done to show how activity of UCH-L5 was affected by b-AP15, while anti-β actin western blot is a loading control. The inactive UCH-L5 has not been detected. The b-AP15 compound inhibited UCH-L5 even at the lower concentration. (**B).** HD11 macrophages were pre-treated with b-AP15 inhibitor (1 µM concentration) or treated with vehicle control (DMSO, added at the same volume as b-AP15). Cells were then infected (inf) with wild-type *Salmonella* Typhimurium or left untreated for 30 minutes. Viability was measured in biological triplicates by Presto Blue Assay according to the manufacturer’s instructions (Life Technologies, USA). The data was analyzed by unpaired Student T test (Prism6, GraphPad Prism Inc., USA).

Supplementary table legend

**S1 Table.** **List of primers used in PCR amplification.** The restriction enzyme digestion sites are represented as the bolded letters, including HindIII (AAGCTT) and EcoRI (GAATTC).

Supplementary methods

Western blotting

HD11 cells were lysed in 0.1% NP-40, 150 mM NaCl, 20 mM CaCl2, 50 mM Tris pH 7.4 containing protease inhibitor cocktail (Roche Applied Science), protein concentration was measured by using the Bio-Rad Protein Assay Kit II (Bio-Rad). The equal amounts of protein from each sample were boiled for 5 minutes at 95°C in 4x reducing sample buffer and separated by SDS-PAGE and subjected to western blotting. The gels were transferred overnight onto Polyvinylidene Fluoride (PVDF) membranes in blotting transfer buffer (25 mM Tris, 190 mM glycine, 20% methanol). Membranes were blocked in Tris-buffered saline (TBS) containing 5% milk and 0.05% Tween 20 for 1 hour, washed in TBS containing 0.05% Tween 20, then incubated with the appropriate primary antibody diluted in 1% milk, 0.05% Tween 20 in TBS for either one hour or overnight at 4°C, washed in TBS containing 0.05% Tween 20 and incubated with HRP-conjugated secondary antibody in TBS containing 1% milk, and 0.05% Tween 20. Luminata Crescendo Western HRP substrate (Millipore, USA) was used for visualization and the blots were exposed to film (Amersham).

Proteomic analysis

Tandem mass spectra were extracted, charge state deconvoluted and deisotoped by Proteome Discoverer version 1.4.0.288. All MS/MS samples were analyzed using Sequest (XCorr) (Thermo Fisher Scientific, San Jose, CA, USA; version 1.4.0.288) and X! Tandem (The GPM, thegpm.org; version CYCLONE (2010.12.01.1)). Sequest (XCorr Only) was set up to search gallus NCBI database (2014, 37118 entries) assuming the digestion enzyme trypsin. X! Tandem was set up to search the same database also assuming trypsin. Sequest (XCorr Only) and X! Tandem were searched with a fragment ion mass tolerance of 0.80 Da and a parent ion tolerance of 1.5 Da. Carbamidomethyl of cysteine was specified in Sequest (XCorr) and X! Tandem as a fixed modification. Glu->pyro-Glu of the n-terminus, ammonia-loss of the n-terminus, gln->pyro-Glu of the n-terminus and oxidation of methionine were specified in X! Tandem as variable modifications. Oxidation of methionine was specified in Sequest (XCorr) as a variable modification. Scaffold (version Scaffold 4.4.1, Proteome Software Inc., Portland, OR) was used to validate MS/MS based peptide and protein identifications. Peptide identifications were accepted if they could be established at greater than 95.0% probability by the Peptide Prophet algorithm with Scaffold delta-mass correction. Protein identifications were accepted if they could be established at greater than 99.0% probability and contained at least 1 identified peptide. Protein probabilities were assigned by the Protein Prophet algorithm. Proteins that contained similar peptides and could not be differentiated based on MS/MS analysis alone were grouped to satisfy the principles of parsimony. The calculated Prophet false discovery date (FDR) was 0.4% for proteins, and 0.67% peptide FDR.

Fisher’s exact test was performed on the replicates to determine whether the observed difference is significant and the p value has been calculated. The data (fold change between spectral counts from infected and uninfected samples) was further visualized as a graph in Excel software.

For the tryptic peptides obtained from a gel band, the search was performed in Proteome Discoverer (ver. 1.4.0.288) using Chicken NCBI database, trypsin as an enzyme, and the maximum missed cleavage site was three. Since the sample was run on LTQ, the precursor mass tolerance and fragment mass tolerance were 1.5 and 1 Da, respectively. Dynamic Modification were Carbamidomethyl / +57.021 Da (C) and Oxidation / +15.995 Da (M). Decoy database search was performed and peptide validator was used. Proteins were grouped, only PSMs with delta Cn better than: 0.1 were considered, and strict maximum parsimony principle was applied. Score versus Charge State filtering was also applied, in which the following minimal SEQUEST (XCorr) scores were considered for respective charge states: Minimal Score for charge state = 1: 1.5, Minimal Score for charge state = 2: 2, Minimal Score for charge state = 3: 2.25, Minimal Score for charge state = 4: 2.5, Minimal Score for charge state = 5: 2.75.

**Sequence alignment analysis**

The deduced amino acid sequences of UCH-L3 and UCH-L5 proteins cloned from HD11 cells were derived by using Lasergene 8 software (DNASTAR, Madison, WI) based on their nucleic acid sequences attained from this study. Other amino acid sequences of UCH-L3 and UCH-L5 proteins from human, cow, mouse, rat, and chicken were obtained from the GenBank database. The ClustalX was used to generate multiple sequence alignments [[16](#_ENREF_16)]. The accession numbers and information of UCH-L3 and UCH-L5 homologs used in the alignment analysis are listed as below: chicken UCH-L3 (NP_990156; *Gallus* *gallus* ubiquitin carboxyl-terminal hydrolase isozyme L3), human UCH-L3-1 (NP_001257881; *Homo* *sapiens* ubiquitin carboxyl-terminal hydrolase isozyme L3 isoform 1), human UCH-L3-2 (NP_005993; *Homo* *sapiens* ubiquitin carboxyl-terminal hydrolase isozyme L3 isoform 2), mouse UCH-L3 (NP_057932; *Mus musculus* ubiquitin carboxyl-terminal hydrolase isozyme L3), rat UCH-L3 (NP_001103635; *Rattus norvegicus* ubiquitin carboxyl-terminal hydrolase isozyme L3), cow UCH-L3 (NP_001035631; *Bos taurus* ubiquitin carboxyl-terminal hydrolase isozyme L3), chicken UCH-L5 (NP_001006530; *Gallus* ubiquitin carboxyl-terminal hydrolase isozyme L5), human UCH-L5-1 (NP_057068; *Homo* *sapiens* ubiquitin carboxyl-terminal hydrolase isozyme L5 isoform 1), human UCH-L5-2 (NP_001186190; *Homo* *sapiens* ubiquitin carboxyl-terminal hydrolase isozyme L5 isoform 2), human UCH-L5-3 (NP_001186191; *Homo* *sapiens* ubiquitin carboxyl-terminal hydrolase isozyme L5 isoform 3), human UCH-L5-4 (NP_001186192; *Homo* *sapiens* ubiquitin carboxyl-terminal hydrolase isozyme L5 isoform 4), mouse UCH-L5-1 (NP_062508; *Mus musculus* ubiquitin carboxyl-terminal hydrolase isozyme L5 isoform 1), mouse UCH-L5-2 (NP_001153338; *Mus musculus* ubiquitin carboxyl-terminal hydrolase isozyme L5 isoform 2), rat UCH-L5 (NP_001012149; *Rattus norvegicus* ubiquitin carboxyl-terminal hydrolase isozyme L5), cow UCH-L5 (NP_776906; *Bos taurus* ubiquitin carboxyl-terminal hydrolase isozyme L5), cow UCH-L5-X1 (XP_005216819; *Bos taurus* ubiquitin carboxyl-terminal hydrolase isozyme L5 isoform X1).

Transient intracellular expression of chicken UCH-L3 and UCH-L5 proteins

The total RNA was isolated from chicken HD11 cells using the RNeasy Mini Kit (Qiagen, Valencia, CA) as manufacture’s protocols. In order to efficiently remove the genomic DNA carried over from RNA isolation procedure, in-tube DNase I digestion method was applied instead of on-column DNase I digestion. The DNase I (Promega, Madison, WI) was added into total RNA sample under the incubation at 37ºC for 20 minutes. The digestion reaction was subsequently cleaned using the RNeasy Mini Kit (Qiagen, Valencia, CA) followed by the cleanup procedure in the manufacture’s manual. The quality of RNA was validated by gel electrophoresis, and the concentration was measured by NanoDrop ND-1000 spectrophotometer (NanoDrop Technologies, Wilmington, DE).

The full-length coding regions of *UCH-L3* and *UCH-L5* were amplified by using RT-PCR. A total of 1.0 µg total RNA was used in reverse transcription reaction via MML-V reverse transcriptase (Promega, Madison, WI) and poly d(T)_15_ primer (Promega, Madison, WI). The gene-specific primers (see Table S1) corresponding to *UCH-L3* (NM_204825) or *UCH-L5* (NM_001006530) transcript were designed based on the nucleic acid sequences published in GenBank database (NCBI) and used to amplify the coding regions of transcripts through PCR using *Pfu* DNA polymerase (Agilent Technologies, Santa Clara, CA). The amplified DNA fragments were cloned into pGEM-T easy vector (Promega, Madison, WI) or pUC19 vector for subsequently sequencing. The coding sequences of *UCH-L3* and *UCH-L5* transcripts were confirmed by using ABI 310 Genetic Analyzer System (Applied Biosystems, Foster City, CA). For each transcript, at least three individual colonies from each cloning event were sequenced for the sequence verification. Due to the sequence difference from NCBI database, the coding region sequence of *UCH-L5* transcript from HD11 cells was further confirmed by additional independent RT-PCR and cloning procedure. The confirmed coding regions of *UCH-L3* and *UCH-L5* transcripts were subsequently sub-cloned into p3xFLAG-CMV-7.1 protein expression vector (Sigma-Aldrich, St. Louis, MO) at *Hind*III and *Eco*RI sites. The large-scale plasmid DNA was purified from 100 ml of *E*. *coli* cell culture containing p3xFLAG-CMV-7.1 with *UCH-L3* or *UCH-L5* recombinant construct, or p3xFLAG-CMV-7.1 vector alone (served as a control) using the EndoFree Plasmid Maxi kit (Qiagen, Valencia, CA) followed by the manufacture’s protocol. The purified plasmid DNAs were then subjected to the transient intracellular protein expression by using Superfect (Qiagen), exactly as described in the manufacturer’s manual to this reagent.

Inflammasome activity studies

The HD11 cells were seeded on 6-well plates (1x10^6^ cells) for 24 hours. The cells were then infected (or not) with *Salmonella* Typhimurium wild-type at MOI 50:1 for 18 hours. The collected cells were resuspended in cell lysis buffer (25mM HEPES, 5mM EGTA, 5mM DTT, pH 7.5) lysed for 10 min. on ice, and centrifuged at 14,000 rpm for 5 min. 50μl of reaction buffer (0.2% CHAPS, 0.2M HEPES, 20% Sucrose, 29mM DTT, pH 7.5) was added to 50ul of cell lysate, followed by addition of 50μM of caspase-1 fluorogenic Z-YVAD-AFC substrate (Enzo Life Sciences, USA) and incubation at 37°C for 1 hr. The fluorescence was read at 400/505 nm Excitation/Emission by using SPECTRAmax M5 (Molecular Devices).
