# Supplementary figures and images for: Activity-Based Proteomic Profiling of Deubiquitinating Enzymes in Salmonella-Infected Macrophages Leads to Identification of Putative Function of UCH-L5 in Inflammasome Regulation
Source: PLoS One. 2015 Aug 12;10(8):e0135531. doi: 10.1371/journal.pone.0135531 (PMC4534353; doi:10.1371/journal.pone.0135531)

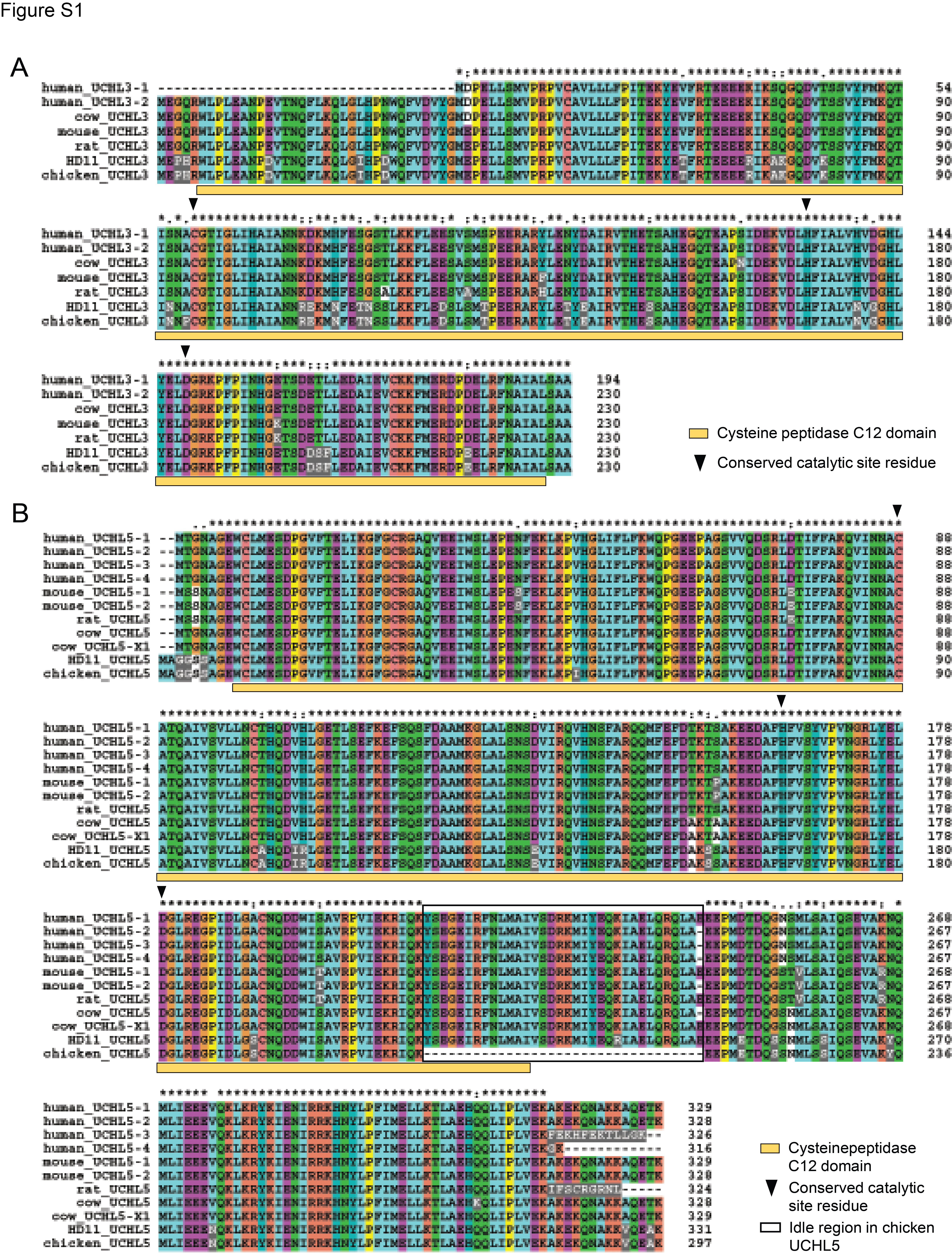

Supplement: S1 Fig — (TIF) [file pone.0135531.s002.tif]

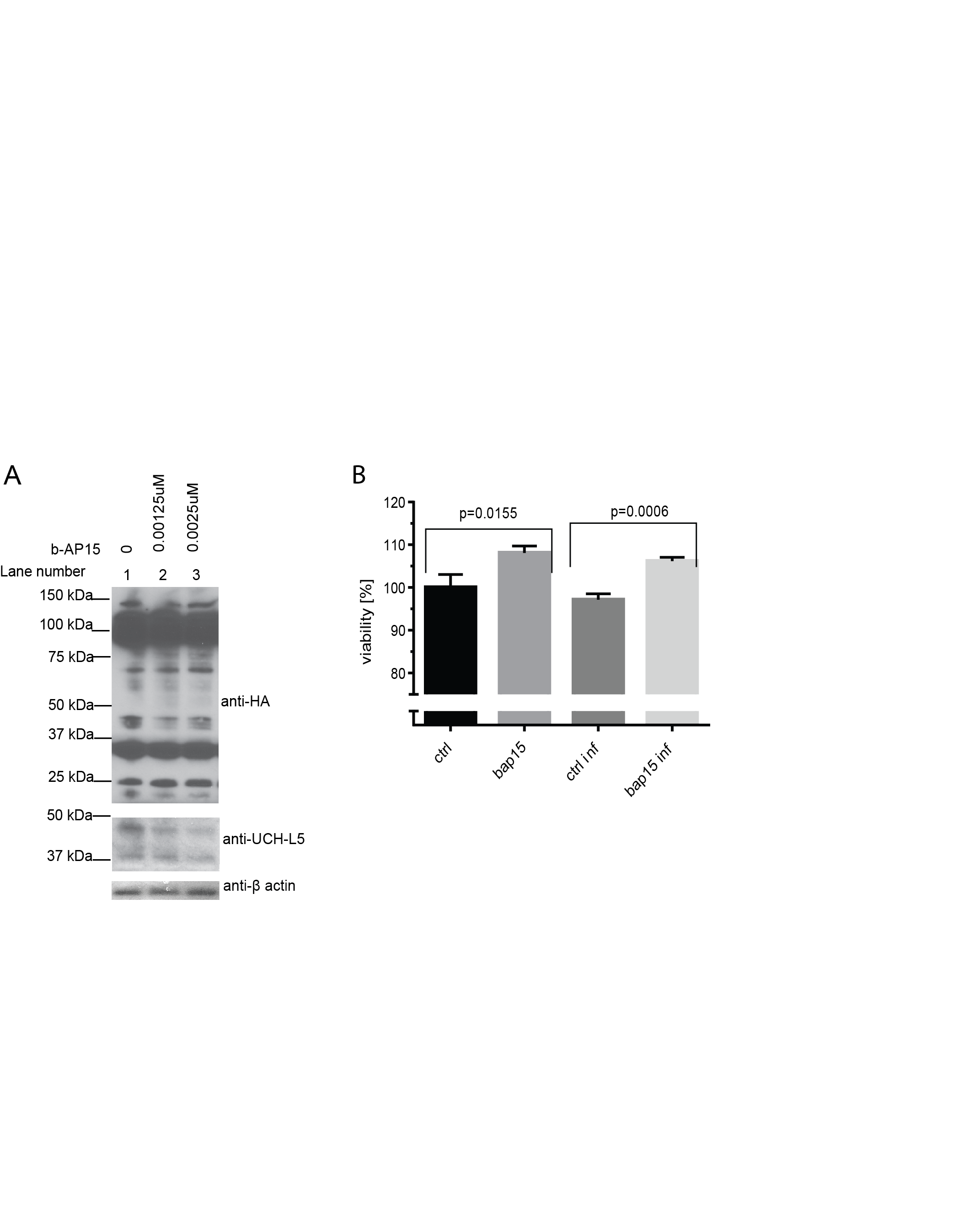

Supplement: S2 Fig — (TIF) [file pone.0135531.s003.tif]
