# Supplementary material for: Activity-Based Proteomic Profiling of Deubiquitinating Enzymes in Salmonella-Infected Macrophages Leads to Identification of Putative Function of UCH-L5 in Inflammasome Regulation
Source: PLoS One. 2015 Aug 12;10(8):e0135531. doi: 10.1371/journal.pone.0135531 (PMC4534353; doi:10.1371/journal.pone.0135531)
Supplement: S1 Table — The restriction enzyme digestion sites are represented as the bolded letters, including HindIII (AAGCTT) and EcoRI (GAATTC). (DOCX) [file pone.0135531.s004.docx]

| Primer Name | Sequence (5’ to 3’) |
| --- | --- |
| UCH-L3-PE-F | GT**AAGCTT**ATGGAGCCGCATCGCTGGCTGCCG |
| UCH-L3-PE-R | GC**GAATTC**TTAAGCTGCAGACAGTGCGATTGC |
| UCH-L5-PE-F | AT**AAGCTT**ATGGCGGGAGGCAGCAGCGCCGGG |
| UCH-L5-PE-R | GC**GAATTC**TCACTTGGCCTCCTGAACTTTCTTG |
